# Pro-inflammatory fatty acid profile and colorectal cancer risk: A Mendelian randomisation analysis

**DOI:** 10.1016/j.ejca.2017.07.034

**Published:** 2017-10

**Authors:** Sebastian May-Wilson, Amit Sud, Philip J. Law, Kimmo Palin, Sari Tuupanen, Alexandra Gylfe, Ulrika A. Hänninen, Tatiana Cajuso, Tomas Tanskanen, Johanna Kondelin, Eevi Kaasinen, Antti-Pekka Sarin, Johan G. Eriksson, Harri Rissanen, Paul Knekt, Eero Pukkala, Pekka Jousilahti, Veikko Salomaa, Samuli Ripatti, Aarno Palotie, Laura Renkonen-Sinisalo, Anna Lepistö, Jan Böhm, Jukka-Pekka Mecklin, Nada A. Al-Tassan, Claire Palles, Susan M. Farrington, Maria N. Timofeeva, Brian F. Meyer, Salma M. Wakil, Harry Campbell, Christopher G. Smith, Shelley Idziaszczyk, Timothy S. Maughan, David Fisher, Rachel Kerr, David Kerr, Michael N. Passarelli, Jane C. Figueiredo, Daniel D. Buchanan, Aung K. Win, John L. Hopper, Mark A. Jenkins, Noralane M. Lindor, Polly A. Newcomb, Steven Gallinger, David Conti, Fred Schumacher, Graham Casey, Lauri A. Aaltonen, Jeremy P. Cheadle, Ian P. Tomlinson, Malcolm G. Dunlop, Richard S. Houlston

**Affiliations:** a Division of Genetics and Epidemiology, The Institute of Cancer Research, London, SW7 3RP, UK; b Genome-Scale Biology Research Program, Research Programs Unit, University of Helsinki, Helsinki, 00014, Finland; c Department of Medical and Clinical Genetics, Medicum, University of Helsinki, Helsinki, 00014, Finland; d Institute for Molecular Medicine Finland (FIMM), University of Helsinki, Helsinki, 00014, Finland; e Wellcome Trust Sanger Institute, Wellcome Trust Genome Campus, Hinxton, Cambridge, CB10 1SA, UK; f Department of Public Health, University of Helsinki, Helsinki, 00014, Finland; g National Institute for Health and Welfare, Helsinki, 00271, Finland; h Folkhälsan Research Centre, Helsinki, 00250, Finland; i Unit of General Practice and Primary Health Care, University of Helsinki and Helsinki University Hospital, Helsinki, 00014, Finland; j Finnish Cancer Registry, Institute for Statistical and Epidemiological Cancer Research, Helsinki, 00130, Finland; k School of Health Sciences, University of Tampere, Tampere, 33014, Finland; l Analytic and Translational Genetics Unit, Department of Medicine, Massachusetts General Hospital, Boston, MA, 02114, USA; m Program in Medical and Population Genetics, The Broad Institute of MIT and Harvard, Cambridge, MA, 02142, USA; n Department of Neurology, Massachusetts General Hospital, Boston, MA, 02114, USA; o Abdominal Center, Department of Surgery, Helsinki University Hospital, Helsinki, 00029, Finland; p Department of Pathology, Central Finland Central Hospital, Jyväskylä, 40620, Finland; q Department of Surgery, Jyväskylä Central Hospital, University of Eastern Finland, Jyväskylä, 40620, Finland; r Department of Genetics, King Faisal Specialist Hospital and Research Center, Riyadh, 12713, Saudi Arabia; s Molecular & Population Genetics Laboratory, Wellcome Trust Centre for Human Genetics, University of Oxford, Oxford, OX3 7BN, UK; t Colon Cancer Genetics Group, University of Edinburgh and MRC Human Genetics Unit, Western General Hospital, Edinburgh, EH4 2XU, UK; u Centre for Population Health Sciences, University of Edinburgh, Edinburgh, EH8 9AG, UK; v Division of Cancer and Genetics, School of Medicine, Cardiff University, Cardiff, CF14 4XN, UK; w CRUK/MRC Oxford Institute for Radiation Oncology, University of Oxford, Oxford, OX3 7DQ, UK; x MRC Clinical Trials Unit, Aviation House, London, WC2B 6NH, UK; y Oxford Cancer Centre, Department of Oncology, University of Oxford, Churchill Hospital, Oxford, OX3 7LE, UK; z Nuffield Department of Clinical Laboratory Sciences, University of Oxford, John Radcliffe Hospital, Oxford, OX3 9DU, UK; aa Department of Epidemiology, Geisel School of Medicine at Dartmouth, Dartmouth-Hitchcock Medical Center, Lebanon, NH, 03756, USA; ab Samuel Oschin Comprehensive Cancer Center, Cedars-Sinai Medical Center, Los Angeles, CA, USA; ac Keck School of Medicine, University of Southern California, Los Angeles, CA, USA; ad Colorectal Oncogenomics Group, Genetic Epidemiology Laboratory, Department of Pathology, The University of Melbourne, Victoria, 3010, Australia; ae Centre for Epidemiology and Biostatistics, The University of Melbourne, Victoria, 3010, Australia; af Department of Health Sciences Research, Mayo Clinic, Scottsdale, AZ, 85259, USA; ag Cancer Prevention Program, Fred Hutchinson Cancer Research Center, Seattle, WA, 98109, USA; ah Lunenfeld-Tanenbaum Research Institute, Mount Sinai Hospital, Toronto, ON, M5G 1X5, Canada; ai Department of Preventive Medicine, University of Southern California, Los Angeles, CA, 90033, USA; aj Center for Public Health Genomics, University of Virginia, Charlottesville, VA, 22908, USA

**Keywords:** Mendelian randomisation, Colorectal cancer, Risk, Plasma fatty acids, Fatty acids

## Abstract

**Background:**

While dietary fat has been established as a risk factor for colorectal cancer (CRC), associations between fatty acids (FAs) and CRC have been inconsistent. Using Mendelian randomisation (MR), we sought to evaluate associations between polyunsaturated (PUFA), monounsaturated (MUFA) and saturated FAs (SFAs) and CRC risk.

**Methods:**

We analysed genotype data on 9254 CRC cases and 18,386 controls of European ancestry. Externally weighted polygenic risk scores were generated and used to evaluate associations with CRC per one standard deviation increase in genetically defined plasma FA levels.

**Results:**

Risk reduction was observed for oleic and palmitoleic MUFAs (OR_OA_ = 0.77, 95% CI: 0.65–0.92, *P* = 3.9 × 10^−3^; OR_POA_ = 0.36, 95% CI: 0.15–0.84, *P* = 0.018). PUFAs linoleic and arachidonic acid had negative and positive associations with CRC respectively (OR_LA_ = 0.95, 95% CI: 0.93–0.98, *P* = 3.7 × 10^−4^; OR_AA_ = 1.05, 95% CI: 1.02–1.07, *P* = 1.7 × 10^−4^). The SFA stearic acid was associated with increased CRC risk (OR_SA_ = 1.17, 95% CI: 1.01–1.35, *P* = 0.041).

**Conclusion:**

Results from our analysis are broadly consistent with a pro-inflammatory FA profile having a detrimental effect in terms of CRC risk.

## 1 Introduction

Colorectal cancer (CRC) is one of the most common cancers and a major cause of cancer-related mortality in economically developed countries [1]. Geographical differences in CRC incidence between countries and migration studies have established the importance of lifestyle and diet as major determinants for CRC risk [2]. Worldwide CRC is currently diagnosed in over one million individuals annually; however, its incidence is set to increase with adoption of western lifestyles in developing countries [3]. Given the importance of diet as a risk factor for CRC, its modification offers the prospect of impacting significantly on disease incidence through public health initiatives.

Dietary fat has been widely implicated as a risk factor for cancer, and meta-analyses of epidemiological studies have tended to associate CRC risk with a higher consumption of red and processed meat [4]. The association between fat intake on cancer risk however, is likely to depend not only on the quantity, but also on the specific type of fatty acid (FA). Animal models and ecological studies have tended to implicate animal fat [5], saturated fatty acid (SFA) and certain omega-6 polyunsaturated fatty acids (ω-6 PUFAs) with an increased risk, and ω-3 PUFA intake with a reduced risk [6], [7], [8]. Evidence for a causal relationship with intake of specific types of fat from epidemiological studies has however largely been inconclusive. Reasons for inconsistencies in observational studies include the inherent problem of eliciting accurate measurements of long-term diet, confounding and reverse causation [9].

Mendelian randomisation (MR) analysis represents an adjunct to the conventional epidemiological observational study for examining associations between an exposure with a disease. The MR strategy makes use of allelic variants that are randomly assigned during meiosis and are robustly associated with traits of interest, as instrumental variables (IVs). Using genetically defined IVs as proxies of modifiable exposure avoids confounding by environmental factors, is not subject to reverse causality and can inform on life-long exposure [10], [11]. Since studies have shown that FA intake influences plasma levels of FAs in theory MR makes an attractive strategy to link dietary FA to CRC risk [12], [13].

We have therefore sought to identify associations between genetically predicted plasma PUFA, MUFAs and SFA levels and CRC risk. Specifically: (1) the ω-6 PUFAs, linoleic acid (LA), arachidonic acid (AA) and dihomo-γ-linolenic acid (DGLA); (2) the ω-3 PUFAs, eicosapentaenoic acid (EPA), docosapentaenoic acid (DPA) and docosahexaenoic acid (DHA); (3) the MUFAs, oleic acid (OA) and palmitoleic acid (POA); and (4) the SFAs, palmitic acid (PA), arachidic acid and stearic acid (SA).

## 2 Methods

### 2.1 Colorectal cancer datasets

We investigated the relationship between genetic risk scores for levels of MUFAs, PUFAs, and SFAs and CRC risk adopting a two-sample MR strategy using data from seven reported genome-wide association studies (GWAS) of CRC (Table 1). Briefly, these GWAS were based on individuals with European ancestry: CCFR1, CCFR2, COIN, FINLAND, UK1, Scotland1 and VQ58 [14]. Each study was approved by respective institutional ethics review board and performed/conducted in accordance with the Declaration of Helsinki.

**Table 1:** Summary of the seven colorectal cancer genome-wide association studies.

Series	Study setting	Study centre	Genotyping platform	No. cases	No. controls
CCFR1	Colon Cancer Family Registry	University of Southern California	Illumina 1M, 1M Duo	1290	1055
CCFR2	Colon Cancer Family Registry	University of Southern California	Illumina 1M, Omni express	796	2236
COIN	COIN trial: Multicentre study of cetuximab and other therapies in metastatic CRC. Controls were unselected blood donors	Cardiff University	Affymetrix Axiom	2244	2162
FINLAND	Finnish Colorectal Cancer Predisposition Study	Helsinki University	Illumina 610K/Illumina HumanOmni2.5M	1172	8266
UK1	CORGI (colorectal Tumour Gene Identification Consortium)	Oxford University	Illumina Hap550	940	965
Scotland1	COGS (Colorectal Cancer Susceptibility Study)	Edinburgh University	Illumina Hap300/240S	1012	1012
VQ58	Cases: VICTOR, post-treatment stages of a phase III, randomised trial of rofecoxib (VIOXX) in patients after potentially curative therapy. QUASAR2, multi-centre study of capecitabine ± bevacizumab as adjuvant treatment. 1958 Birth cohort controls	Oxford University	Illumina Hap300/370, Illumina 1M	1800	2690

### 2.2 Genotyping data

Comprehensive details of the genotyping and quality control of the seven GWAS have been previously reported [14]. Briefly, we excluded single nucleotide polymorphisms (SNPs) with a minor allele frequency of <1%, low call rate <95%, those SNPs violating Hardy–Weinberg equilibrium, and individuals with non-European ancestry as assessed using data from HapMap v2 [15]. IMPUTEv2 software [16] was used to recover untyped SNP genotypes using a merged reference panel consisting of Sequencing Initiative Suomi (for the FINLAND data) or UK10K (for the remaining data) and 1000 Genomes Project data [17], [18]. Poorly imputed SNPs, defined by an INFO score of <0.9, were excluded. Summary statistics from the seven GWAS were used to calculate the odds ratios (ORs) for FA-related SNPs.

### 2.3 Gene variants used to construct genetic risk scores

Genetic risk scores for IVs for each plasma FA were developed from SNPs previously identified by The Cohorts for Heart and Aging Research in Genomic Epidemiology (CHARGE) Consortium. We considered SNPs associated at genome-wide significance (*i.e. P* ≤ 5.0 × 10^−8^) in individuals with European Ancestry. To avoid co-linearity between SNPs for each FA we imposed a threshold *r*^2^ value of ≥0.01 for linkage disequilibrium (LD) including only the SNPs with the strongest effect on the trait in genetic risk scores (Table 2, [19], [20], [21], [22]). For each identified SNP, we recovered the chromosome positions, the risk alleles, association estimates and standard errors. For each SNP, the allele that was associated with increased FA level was considered the effect allele.

**Table 2:** Effect sizes for plasma fatty acid content (per standard deviation increase in levels) for genome-wide significant (*P* < 5 × 10^−8^) instrumental variables reported by CHARGE consortium.

FA subtype	Fatty acid	SNP ID	Chr	Position (bp)^a^	Allele	β	StdErr	*P*-value	Variance explained^b^
SFA	Arachidic acid (20:0)	rs680379	20	12917400	**A**/G	0.098	0.01	5.81 × 10^−13^	–
	Palmitic acid (PA) (16:0)	rs2391388	1	95485825	**C**/A	0.18	0.03	2.72 × 10^−11^	0.21–0.98%
	Stearic acid (SA) (18:0)	rs6675668	1	95515637	**G**/T	0.17	0.02	2.16 × 10^−18^	0.37–1.39%
		rs11119805	1	211918244	**T**/A	0.17	0.03	2.8 × 10^−09^	<0.01–0.72
		rs102275	11	61557803	**T**/C	0.18	0.02	1.33 × 10^−20^	0.33–1.34%
ω-3 PUFA	Docosahexaenoic acid (DHA) (22:6n-3)	rs2236212	6	10995015	**G**/C	0.11	0.01	1.26 × 10^−15^	0.7%
	Docosapentaenoic acid (DPA) (22:5n-3)	rs780094	2	27741237	**T**/C	0.02	0.003	9.04 × 10^−09^	–
		rs3734398	6	10982973	**C**/T	0.04	0.003	9.71 × 10^−43^	8.6%
		rs174547	11	61570783	**T**/C	0.07	0.003	3.79 × 10^−154^	2.8%
	Eicosapentaenoic acid (EPA) (20:5n-3)	rs3798713	6	11008622	**C**/G	0.035	0.005	1.93 × 10^−12^	0.4%
ω-6 PUFA	Arachidonic acid (AA) (20:4n-6)	rs174547	11	61570783	**T**/C	1.69	0.03	3.30 × 10^−971^	3.7–37.6%
		rs16966952	16	15135943	**G**/A	0.2	0.03	2.43 × 10^−10^	0.1–0.6%
	Dihomo-γ-linolenic acid (DGLA) (20:3n-6)	rs174547	11	61570783	**C**/T	0.36	0.01	2.63 × 10^−151^	8.7–11.1%
		rs16966952	16	15135943	**G**/A	0.22	0.02	7.55 × 10^−65^	2.0–4.5%
	Linoleic acid (LA) (18:2n-6)	rs10740118	10	65101207	**G**/C	0.25	0.04	8.08 × 10^−09^	0.2–0.7%
		rs174547	11	61570783	**C**/T	1.47	0.04	4.98 × 10^−274^	7.6–18.1%
		rs16966952	16	15135943	**A**/G	0.35	0.04	1.23 × 10^−15^	0.5–2.5%
ω-7 MUFA	Palmitoleic acid (POA) (16:1n-7)	rs780093	2	27742603	**T**/C	0.02	0.003	9.80 × 10^−10^	0.23–0.93%
		rs6722456	2	134529091	**G**/A	0.05	0.009	4.12 × 10^−08^	<0.01–0.57
		rs603424	10	102075479	**G**/A	0.03	0.004	5.69 × 10^−15^	0.28–1.57%
		rs11190604	10	102302457	**G**/A	0.02	0.004	5.69 × 10^−09^	0.02–0.71%
		rs102275	11	61557803	**C**/T	0.02	0.003	6.60 × 10^−13^	0.15–1.03%
ω-9 MUFA	Oleic acid (OA) (18:1n-9)	rs102275	11	61557803	**C**/T	0.23	0.02	2.19 × 10^−32^	0.32–2.14%

FA, fatty acid; SNP, single nucleotide polymorphism; bp, base pair; SFA, saturated fatty acid; MUFA, monounsaturated fatty acid; PUFA, polyunsaturated fatty acid; StdErr, standard error. Effect allele influencing each FA trait is marked in bold.

a hg19 NCBI build.

b Taken from CHARGE consortium, as a percentage of total serum fatty acids, calculated by (β^2^*2*MAF*(1-MAF))/Var(Y) where β is the regression coefficient, MAF is the minor allele frequency and Var(Y) is the variance in levels of the fatty acid. IVs obtained from Refs. [19], [20], [22].

### 2.4 Statistical analysis

The association between the plasma level of each FA and CRC was examined using MR on summary statistics as per Burgess (2015) [23]. The ratio estimate $\left(\hat{\beta }\right)$ of all SNPs associated with each fatty acid, combined, on CRC was calculated as follows:

$$\hat{\beta }=\frac{\sum_{k}X_{k}Y_{k}\sigma_{Y_{k}}^{-2}}{\sum_{k}X_{k}^{2}\sigma_{Y_{k}}^{-2}}\text{.}$$

where *X*_*k*_ corresponds to the association of SNP *k* (as log of the OR per risk allele) with the fatty acid trait *Y*, *Y*_*k*_ is the association between SNP *k* and CRC risk (as log of the OR) with standard error $\sigma_{Y_{k}}$. The estimate for $\left(\hat{\beta }\right)$ represents the causal increase in the log odds of the CRC, per unit change in fatty acids. The standard error of the combined ratio estimate is given by:

$$\mathrm{se}\left(\hat{\beta }\right)=\;\sqrt{\frac{1}{\sum_{k}X_{k}^{2}\sigma_{Y_{k}}^{-2}}}\text{.}$$

A meta-analysis of statistics for each specific FA generated for each CRC cohort was combined under fixed-effects models to derive the summary ORs and confidence intervals (CIs). To assess the impact of between study heterogeneity, we also derived ORs under a random-effects model.

A central tenet in MR is the absence of pleiotropy (*i.e.* a gene influencing multiple traits) between the SNPs influencing CRC risk and FA levels. This would be revealed as deviation from a linear relationship between SNPs and their effect size for any FA and CRC risk. To examine for violation of the standard IV assumptions in our analysis, we performed inverse variant weighted (IVW) and MR-Egger regression tests [24].

We considered a significance level of *P* ≤ 0.05 as being satisfactory to derive a conclusion. While ordinarily it would be appropriate to impose a Bonferroni-corrected threshold, this assumes an independence of IVs across all FA traits, which is not the case in the present analysis. All statistical analyses were undertaken using R version 3.1 software [25].

### 2.5 Expression quantitative trait locus analysis

To examine the relationship between SNP genotype and expression of FA metabolism genes, we performed expression quantitative trait locus (eQTL) analysis using data from The Cancer Genome Atlas (TCGA) and the genotype tissue expression (GTEx)project [26], [27].

## 3 Results

**Fig. 1:**
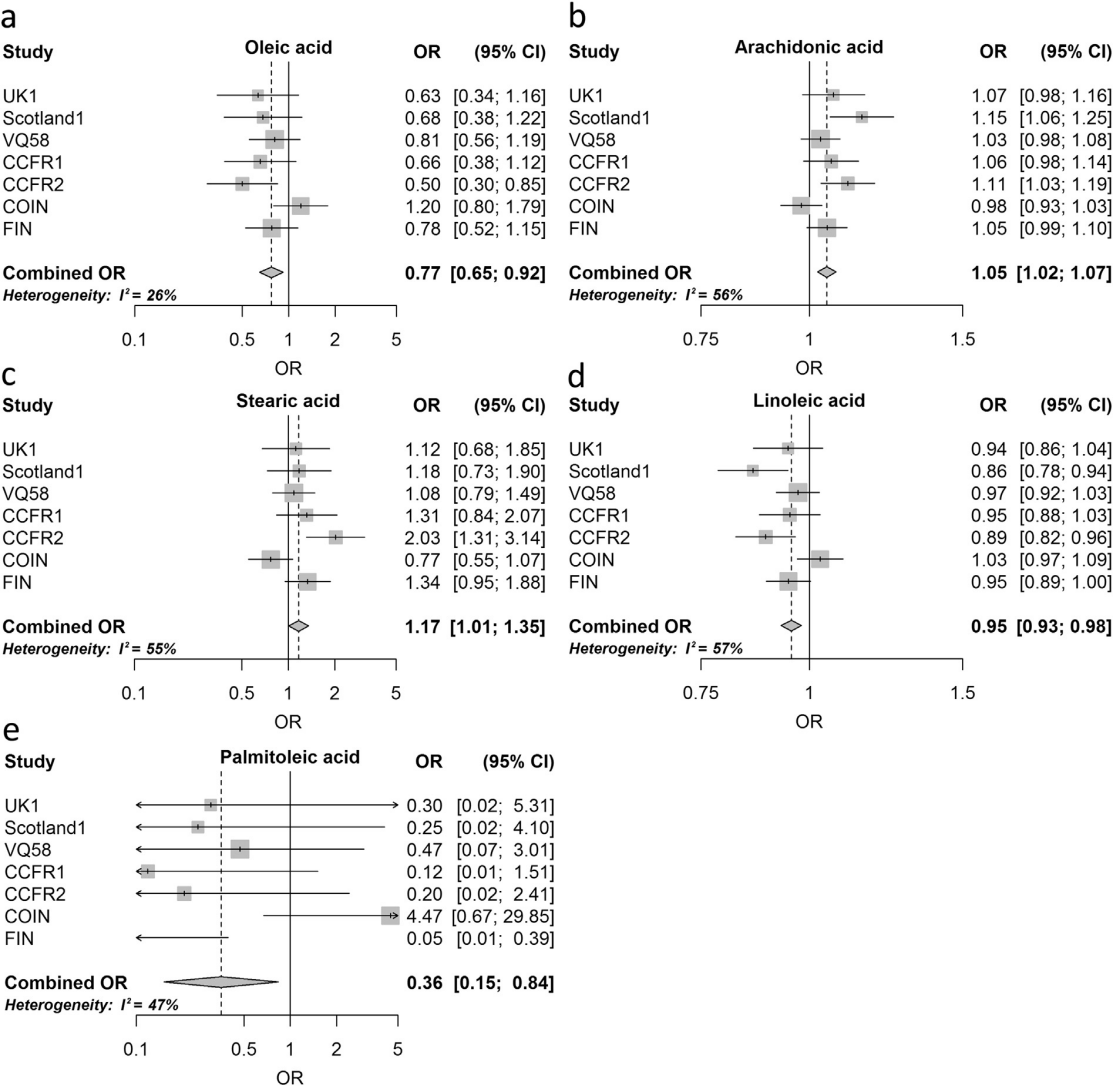
Meta-analysis odds ratios (OR) for colorectal cancer per unit increase in genetic risk score (standard deviation of trait) for significant fatty acid associations. (a) Oleic acid; (b) arachidonic acid; (c) stearic acid; (d) linoleic acid; (e) palmitoleic acid; *I*^2^: proportion of the total variation due to heterogeneity. Boxes: OR point estimate; its area is proportional to the weight of the study. Diamond: overall summary estimate, with confidence intervals given by its width. Vertical line: null value (OR = 1.0).

The FA-associated genetic variants and their GWAS-reported characteristics that were used to derive IVs for FAs are detailed in Table 2. A reduced risk of CRC was observed for genetic variants associated with increases in the MUFAs studied (Table 3). In all but one of the seven cohorts increased levels of OA were associated with reduced CRC risk (Fig. 1). In the meta-analysis of these seven cohorts the OR_OA_ was 0.77 (95% CI: 0.65–0.92, *P* = 3.9 × 10^−3^) with little evidence of between-study heterogeneity (*P*_het_ = 0.23, *I*^2^ = 26%). Similarly, increased levels of POA were associated with reduced CRC risk with an OR_POA_ of 0.36 (95% CI: 0.15–0.84, *P* = 0.018, *P*_het_ = 0.08, *I*^2^ = 47%; Fig. 1).

**Table 3:** Odds ratios (ORs) and 95% confidence intervals (CI) for one standard deviation increase in genetically predicted plasma fatty acid levels and colorectal cancer risk.

Fatty acid	Significant associations							
	OR (fixed effects)	95% CI (fixed effects)	*P*-value (fixed effects)	OR (random effects)	95% CI (random effects)	*P*-value (random effects)	*I*^2^	*P*_het_
Arachidic acid	0.92	0.61–1.39	0.7	0.93	0.61–1.40	0.71	3%	0.41
Palmitic acid (PA)	0.97	0.78–1.21	0.82	0.97	0.78–1.21	0.82	0%	0.47
Stearic acid (SA)	1.16	1.01–1.35	0.04	1.2	0.95–1.49	0.12	55%	0.04
Docosahexaenoic acid (DHA)	1.32	0.94–1.87	0.11	1.32	0.94–1.87	0.11	0%	0.65
Docosapentaenoic acid (DPA)	1.58	0.99–2.52	0.06	1.63	0.97–2.73	0.06	17%	0.3
Eicosapentaenoic acid (EPA)	0.39	0.13–1.21	0.1	0.39	0.13–1.21	0.1	0%	0.57
Arachidonic acid (AA)	1.05	1.02–1.07	1.7 × 10^−4^	1.05	1.02–1.09	4.9 × 10^−3^	56%	0.03
Dihomo-γ-linolenic acid (DGLA)	0.91	0.83–1.00	0.06	0.95	0.80–1.01	0.07	23%	0.26
Linoleic acid (LA)	0.95	0.93–0.98	3.7 × 10^−4^	0.95	0.91–0.99	8.9 × 10^−3^	57%	0.03
Oleic acid (OA)	0.77	0.65–0.92	3.9 × 10^−3^	0.76	0.62–0.94	9.7 × 10^−3^	26%	0.23
Palmitoleic acid (POA)	0.36	0.15–0.84	0.018	0.32	0.10–1.07	0.06	47%	0.08

*P*_het_, *P*-value for heterogeneity; *I*^2^, proportion of the total variation due to heterogeneity; SFA, saturated fatty acid; PUFA, polyunsaturated fatty acid; MUFA, monounsaturated fatty acid.

The ω-6 PUFAs LA and AA both showed association with CRC risk, but in different directions. Specifically, LA was associated with reduced risk (OR_LA_ = 0.95, 95% CI: 0.93–0.98, *P* = 3.7 × 10^−4^, *P*_het_ = 0.03, *I*^2^ = 57%; Fig. 1) and AA with an increased risk (OR_AA_ = 1.05, 95% CI: 1.02–1.07, *P* = 1.7 × 10^−4^, *P*_het_ = 0.03, *I*^2^ = 56%). The association between one standard deviation increase in each of the other PUFAs defined by their respective IVs and CRC risk were null (Supplementary Fig. 1).

Of the three SFAs studied, increased SA was nominally associated with CRC risk (OR_SA_ = 1.17, 95% CI: 1.01–1.35, *P* = 0.041, *P*_het_ = 0.04, *I*^2^ = 55%).

To formally assess the impact of heterogeneity on study findings we derived ORs under a random-effects model. Associations between AA, LA and OA and CRC risk remained significant (Table 3).

We assessed the impact of possible classical pleiotropism on MR estimates using both IVW and MR-Egger regression tests. There was no evidence for violation of the standard IV assumptions used for MR analysis, such as a dependence on confounders (Table 4).

**Table 4:** IVW and MR-Egger test results for combined fatty acid instrumental variables.

Fatty acid subtype	Fatty acid	IVW		MR-Egger		
		Slope Estimate (95% CI)	*P*-value		Estimate (95% CI)	*P*-value
SFA	Stearic acid (SA)	−0.1 (−0.33 to 0.64)	0.30	Intercept	−0.68 (−4.79 to 3.43)	0.28
				Slope	4.10 (−19.86 to 28.06)	0.27
ω-3 PUFA	Docosapentaenoic acid (DPA)	0.46 (−2.32 to 3.23)	0.55	Intercept	−0.09 (−0.56 to 0.39)	0.26
				Slope	2.01 (−7.9 to 11.61)	0.23
	Eicosapentaenoic acid (EPA)	−0.59 (−7.99 to 9.16)	0.54	Intercept	−0.11 (N/A)	–
				Slope	2.2 (N/A)	–
ω-6 PUFA	Arachidonic acid (AA)	0.04 (−0.2 to 0.33)	0.29	Intercept	0.04 (N/A)	–
				Slope	0.02 (N/A)	–
	Dihomo-γ-linolenic acid (DGLA)	−0.09 (−2.48 to 2.29)	0.70	Intercept	0.25 (N/A)	–
				Slope	−0.90 (N/A)	–
	Linoleic acid (LA)	−0.05 (−0.17 to 0.07)	0.22	Intercept	0.02 (−0.64 to 0.67)	0.77
				Slope	−0.07 (−0.81 to 0.68)	0.46
MUFA	Palmitoleic acid (POA)	−1.03 (−2.64 to 0.58)	0.15	Intercept	−0.11 (−0.27 to 0.05)	0.12
				Slope	3.13 (−3.16 to 9.41)	0.21

CI, confidence interval; MUFA, monounsaturated fatty acid; PUFA, polyunsaturated fatty acid; SFA, saturated fatty acid; IVW, inverse variant weighted. *FA traits with two IVs, preventing calculation of CIs and *P*-value.

**Fig. 2:**
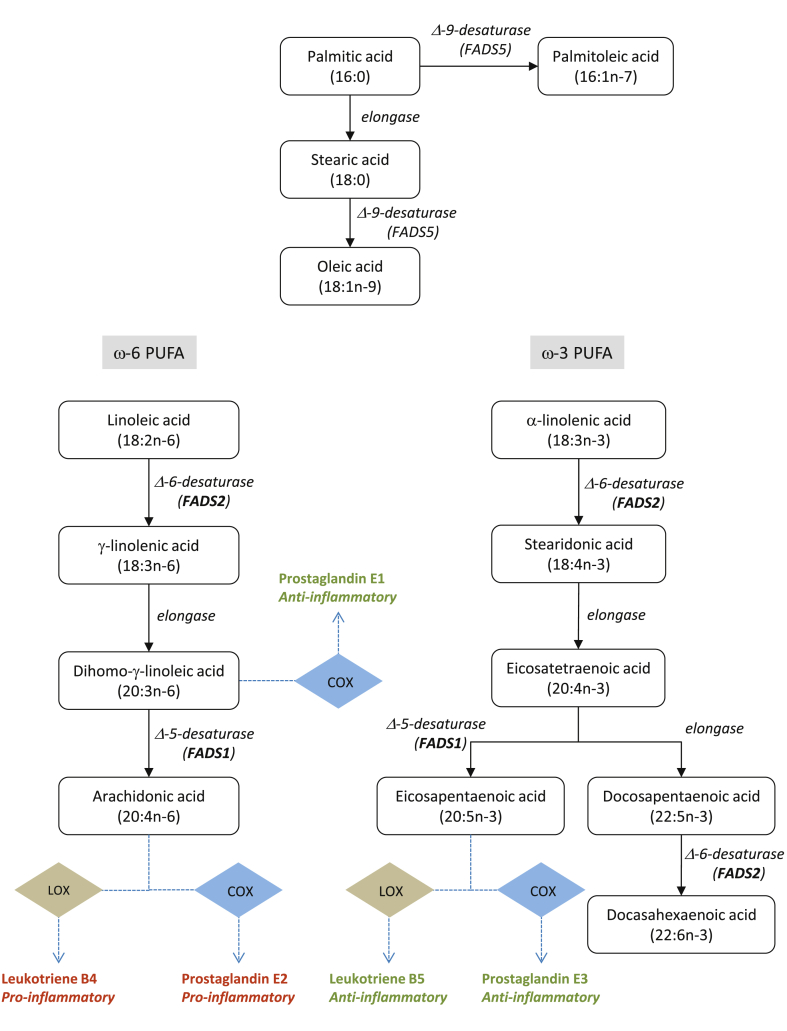
Pathway of fatty acids. Shown are the various fatty acids analysed, and the enzymes involved in their metabolism. COX: cyclooxygenase, LOX: 5-lipoxygenase.

In the present analysis, we used the SNP rs102275 in combination with other SNPs to generate a polygenic risk score for SA, OA and POA, whereas rs174547, which is in LD with rs102275 (*r*^2^ = 1.0 and D′ = 1.0), was used for DPA, AA, DGLA and LA. Both SNPs annotate the *FADS2* gene. FADS2 is a rate-limiting enzyme in the desaturation of LA to AA, and α-linolenic acid into DHA and EPA (Fig. 2). These FAs are precursors for prostaglandins and leukotrienes, which are key mediators of the inflammatory response. In an eQTL analysis rs174547 and rs102275 genotype were shown to be strongly correlated with *FADS2* expression across a range of different tissue types, including blood (*P* = 3.98 × 10^−29^), normal colon (*P* = 1.65 × 10^−10^) and CRC (*P* = 2.07 × 10^−5^) (Supplementary Table 1).

## 4 Discussion

While dietary fat intake has been associated with the CRC risk, teasing out specific FA associations and their mechanistic basis has proven to be challenging. A number of observational studies have reported associations between serum levels of specific FAs with CRC [28], [29], supporting our findings.

A major strength of the MR strategy to identify causal associations is that it is not influenced by recall bias and confounding that can affect traditional observational studies. Nevertheless, a key assumption in MR is that the variants used to generate genetic scores are associated with the exposure being queried. Herein, we only made use of SNPs associated with each FA at genome-wide significance from hypothesis-free GWAS. Furthermore, we only used data from individuals of European descent so as to limit bias from population stratification. Another central assumption in MR is that variants are associated with CRC only through the exposure and are not confounded by pleiotropy, which would be revealed by a positive correlation between increasing effect sizes in the IVs and CRC risk. While we did not observe such relationship, we acknowledge that IVs for a number of the FAs were solely based on only one or two SNPs, preventing assessment by IVW and MR-Egger analysis. One strategy to overcome this and fully investigate any pleiotropy would be to measure FA serum levels in correlation with CRC risk.

In this analysis, the same SNP (rs102275, or correlated SNP rs174547) was used to make causal deductions between multiple FAs and CRC risk. Therefore, SNPs have been used each time assuming that the exposure individually accounts for the disease association. The genetic variant association with CRC risk is consequently double-counted, in that the effect is attributed to different FA exposures [30]. With such vertical pleiotropism, single locus MR analyses cannot robustly decipher which FA is primarily driving the relationship with CRC risk. Such considerations have not been addressed in previous studies of the relationship between PUFAs and prostate cancer [31] or between branched-chain amino acids and diabetes [32].

While we did not demonstrate a causal association between other FAs including several PUFAs, SFAs and CRC risk, we acknowledge that our power to demonstrate a relationship was limited. For example, with respect to EPA: assuming the variance explained by the alleles is 0.04%, based on epidemiological observational study data, and a relative risk of 1.04 we had <10% power to demonstrate a relationship [33].

Accepting these caveats we have provided support for differing effects of OA, and ω-6 PUFAs LA and AA on CRC risk. Our findings broadly accord with the findings from many of the published ecological and epidemiological observational studies. Notably, increased levels of AA contribute as a risk factor to CRC development [34], [35], while increased intake of olive oil, which is high in OA, is associated with decreased risk [36]. A number of epidemiological studies have provided evidence that a Mediterranean diet, with a higher olive oil intake, is associated with reduced CRC risk [36], [37], [38].

In the eQTL analysis, both rs102275 and rs174547 show evidence of *cis*-regulatory effects on *FADS2* expression. Intriguingly, rs174547 has previously been reported to have opposing effects on *FADS2* and *FADS1* expression in CRC [39]. Collectively, these data provide for relationship between diet, genotype, FA metabolism and CRC risk through modulation of an inflammatory response.

Even so, a biological basis for associations between specific FAs and CRC risk remain to be established. It is however, predicted *a priori* that within any FA class, different members have different actions and effects. With respect to ω-6, evidence supports the inflammatory effects for AA through COX-2 production of inflammatory mediators [40] including prostaglandin E2, which affect CRC carcinogenesis [41], [42], [43]. This implies that diets high in AA, such as meat or eggs, may lead to more inflammatory compounds, which in turn may increase CRC risk. While increasing dietary LA, an essential FA, might potentially enrich tissues with AA due to their metabolic link [44], a gene–environment interaction may exist to influence colon FA content [45]. There is however, contradictory evidence from studies that have associated LA with both an increased [46] and decreased risk of CRC, possibly by altering ω-6 to ω-3 FA ratios [47] or alternatively production of reactive oxygen species [48]. The ability of aspirin to irreversibly inhibit COX-1 and COX-2 and therefore lower pro-inflammatory signals independent of genotype and diet, has thus proved an attractive option for CRC chemoprevention [49].

In conclusion, irrespective of the biological basis of associations between FAs and CRC risk our findings are consistent with the observation that the dietary composition of MUFAs in Mediterranean diets are risk reducing, and that a pro-inflammatory diet are risk increasing [50]. While we may not be at a stage where we can justifiably advise individuals to alter their intake of specific FAs to decrease the risk of developing CRC, it seems the current guidelines to moderate total fat and SFA consumption and increase unsaturated FA intake is likely to be beneficial.

## Conflict of interest statement

None declared.
