# Supplementary material for: Pro-inflammatory fatty acid profile and colorectal cancer risk: A Mendelian randomisation analysis
Source: Eur J Cancer. 2017 Oct;84:228–38. doi: 10.1016/j.ejca.2017.07.034 (PMC5630201; doi:10.1016/j.ejca.2017.07.034)
Supplement: Supplementary file 1 [file mmc1.pdf]

Supplementary Material

Figure 1: Meta-analysis odds ratios (OR) for colorectal cancer per unit increase in genetic risk score (standard deviation of trait) for non-significant fatty acid associations.

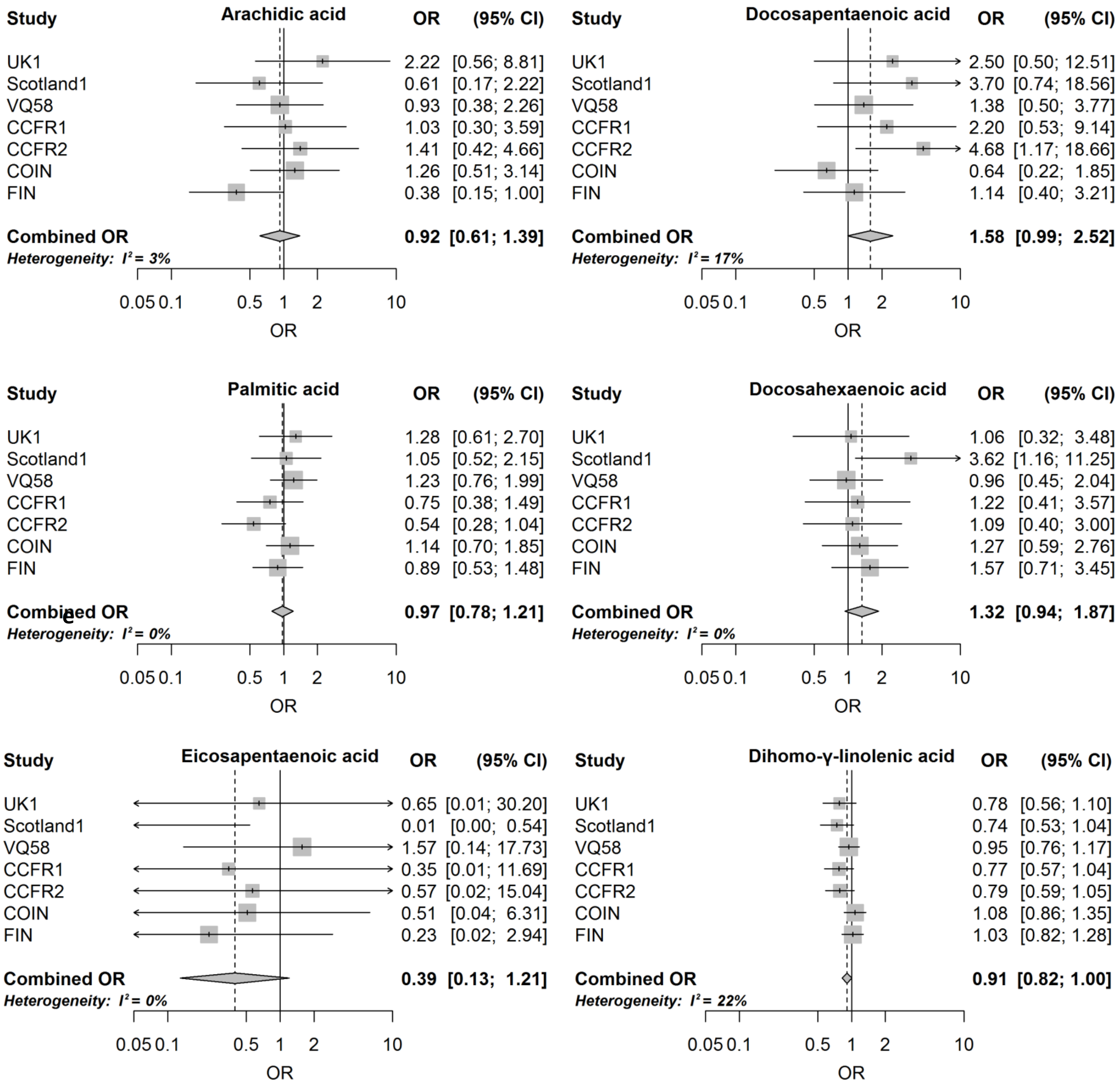

I^2, proportion of the total variation due to heterogeneity. Boxes: OR point estimate; its area is proportional to the weight of the study. Diamond, overall summary estimate, with confidence intervals given by its width. Vertical line: null value (OR = 1.0).
